# Diagnostics challenges and therapeutic response in Blau syndrome – case report

**DOI:** 10.1186/1546-0096-12-S1-P272

**Published:** 2014-09-17

**Authors:** Luciana B Paim-Marque, Leda Azevedo, Átila Botelho Ponte, Marcelo Lima, Melina Teófilo, Fernanda Honório

**Affiliations:** 1 Pediatric rheumathology, Hospital Infantil Albert Sabin, Fortaleza, Brazil; 2 Medicine - Pediatric Rheumathology, Fortaleza university, Fortaleza, Brazil; 3 Medicine - Pediatric Rheumathology, Hospital Infantil Albert Sabin, Fortaleza, Brazil

## Introduction

Blau’s syndrome is an autosomal-dominant, autoinflammatory disease characterized by a non caseous granulomatous inflammation,presenting with arthritis, dermatitis and uveitis, caused by mutations of the *CARD15/NOD2*. Patients are treated with high doses of oral corticosteroids,and if the therapeutic response is unsatisfactory,additional treatment with immunosuppressive agents is the best choice,such as: Methotrexate,Cyclosporine,Anti-TNF and Canakinumab (Anti-IL1).

## Objectives

Describe a case of Blau syndrome with early onset atypical manifestations and therapeutic response.

## Methods

### Case Report

A 2 year old boy was referred to a brazilian Pediatric Rheumatology center, in march/2009, with daily persistent high fever, diffuse and firm erythematous rash and polyarthritis since age of 3 months old. Laboratory tests showed: leukocytosis, anaemia and persistent elevation of platelets count; Immunoglobulins, complements; Zinc,Calcium, Phosphorus, PTH, 25-hidroxivitamin D e Urine analysis: normal. Cytomegalovirus, Epstein Barr, toxoplamosis, Parvovirus B19, HIV, herpes, Auto-antibodies (FAN, DNA, SSa, SSb, RNP, Sm, ANCA-p, ANCA-c, anticardiolipine e lupus anticoagulant): negative. VHS e PCR: elevated; PPD: nonreactive. Audiometry /Impedanciometry: normal. Wrist X-Ray: nodular images in 2nd and 5th right metacarpals. Skin and synovial biopsy: granulomatous alterations. His mother presented similar history, diagnosed “systemic JIA” at age of 6, currently presenting articular deformities and visual loss. Indomethacin, cyclosporine, methotrexate, colchicine, etanercept and adalimumabe,were used but the patient remained with fever, rash and worsening of articular symptoms; ophtalmopathy in left eye and left ventricular dysfunction. However, there were no signs of neurological involvement. Canakinumab (Anti IL1) was started with improvement of all the symptoms. The genetic test detected genetic mutation in NOD2.

## Results

Systemic JIA was initially considered. However, there were no significant improvement after treatment with prednisone, cyclosporine, NSAIDs and Methotrexate. Considering the very early onset, CINCA syndrome, caused by mutation of gene CIAS1, was also an important hypothesis. The manifestations of high fever and urticarial rash starting in first weeks of life; asseptic meningitis leading to sensorineural hearing and vision loss with persistent elevation of acute phase reactants, leukocytosis and chronic anaemia. In spite of the clinical resemblance, cutaneous granulomatosis and the lack of neurological symptoms rule out this diagnosis.Finally with the noncaseating granulomatosus skin biopsy, we considered Blau’s syndrome. It usually manifests before the first decade of life, with small and big joint symmetric arthritis, variable erythematous rash (maculopapular or icthyosiform) and uveitis. The early onset, the skin rash and the unsatisfactory response to the initial treatment brought us some doubts. But the gene mutation in NOD2/CARD15 was conclusive. Blau’s syndrome can be treated initially with NSAIDs and systemic corticosteroids, however, in cases with ineffective therapeutic response Anti-TNF and Anti-IL1 can be very beneficial, especially in patients presenting non responsive uveitis.

## Conclusion

The atypical manifestation of this case shows the variable clinical spectrum of monogenic autoinflammatory syndromes and their resemblances, which makes the early diagnosis and treatment very challenging.

## Disclosure of interest

L Paim-Marque Paid Instructor for: Novartis, Shire, L Azevedo: None declared, ÁB Ponte: None declared, M Lima: None declared, M Teófilo: None declared, F Honório: None declared.

